# Assessing the adaptive role of cannabidivarinic acid (CBDVA) in aphid defense in *Cannabis sativa*

**DOI:** 10.1186/s42238-025-00291-x

**Published:** 2025-06-11

**Authors:** Jacob MacWilliams, Venkatesh Padimi, Olivia Carter, Korey Brownstein, Zachary Stansell, Tyler Gordon, Punya Nachappa

**Affiliations:** 1 Department of Agricultural Biology, Colorado State University, Fort Collins, Colorado 80523 U.S.A.; 2 USDA, ARS, National Center for Agricultural Utilization Research, Peoria, Illinois 61604 U.S.A.; 3 Plant Genetic Resources Unit, USDA, ARS, Geneva, New York 14456 U.S.A.; 4 Horticulture Section, School of Integrative Plant Science, Cornell University, Geneva, NY 14456 U.S.A.

**Keywords:** Hemp, Cannabis aphids, Green peach aphids, Cannabinoids, CBD, Pest management

## Abstract

**Background:**

*Cannabis sativa* has unique secondary metabolites known as cannabinoids, which include tetrahydrocannabinol (THC) and cannabidiol (CBD) and more than 100 related secondary metabolites. There is increasing evidence that cannabinoids can affect insect fecundity and survival. In this study, we assessed the role of a minor cannabinoid, cannabidivarinic acid (CBDVA) on fecundity and survival of *C. sativa*-adapted specialist aphid, cannabis aphid (*Phorodon cannabis*) and non-adapted, generalist aphid, green peach aphid (*Myzus persicae*).

**Methods:**

We evaluated a panel of high and low-CBDVA hemp genotypes obtained from the USDA-ARS Hemp Germplasm Collection at the Plant Genetic Resources Unit for cannabis aphid resistance in greenhouse experiments. Trichome measurements were recorded for genotypes with the highest and lowest aphid counts. To confirm the role of CBDVA, we performed artificial feeding assays by supplementing CBDVA in aphid diets in the laboratory.

**Results:**

We found that cannabis populations were significantly higher (Mean ± SE: 221.57 ± 37.27) on a low-CBDVA genotype compared to a high-CBDVA genotype (12.58 ± 3.53) after 14 days of aphid infestation. The high-CBDVA genotype had significantly more trichomes than the low-CBDVA genotype. Supplementation of CBDVA in artificial diets decreased cannabis aphid fecundity from 109.56 ± 10.01 nymphs on diet control and 52.67 ± 7.79 nymphs on DMSO control to 18.71 ± 5.21 nymphs on 1 mM CBDVA + DMSO supplementation after 4 days. CBDVA + DMSO supplementation decreased green peach aphid fecundity from 72.36 ± 6.82 on diet control and 72.50 ± 3.97 on DMSO control to 11.60 ± 2.60 on 0.5 mM CBDVA after 3 days.

**Conclusions:**

Our results show that CBDVA has insecticidal activity against cannabis aphids and green peach aphids. CBDVA’s potential as a pure essential oil may be an environmentally sustainable pest management option for organic production systems.

**Supplementary Information:**

The online version contains supplementary material available at 10.1186/s42238-025-00291-x.

## Background

Plant responses to insect attack involve the activation of multiple signaling pathways that produce secondary or specialized metabolites that have toxic, repellent, and/or anti-nutritional effects on herbivores (Howe and Jander 2008; War et al. 2012). *Cannabis sativa* L. is well-known for producing cannabinoids, which include Δ9-tetrahydrocannabinol (THC) and cannabidiol (CBD), but more than 750 secondary metabolites have been identified from *C. sativa*, including over 100 different cannabinoids (Andre et al. 2016; Laaboudi et al. 2024). One hypothesis is that cannabinoids evolved as novel herbivory defense adaptations (Rothschild et al. 1977; Rothschild and Fairbairn 1980; McPartland 1997). Previous research showed that garden tiger moths (*Arctia caja*) feeding on high-THC cannabis plants were stunted and unable to survive past third instars, and when THC was sprayed on cabbage (*Brassica oleracea*), it deterred oviposition of the large white (*Pieris brassicae*) (Rothschild et al. 1977; Rothschild and Fairburn, 1980). More recent studies have demonstrated the negative impact of CBD on tobacco hornworm (*Manduca sexta*) (Park et al. 2019) and fall armyworm (*Spodoptera frugiperda*) (Abendroth et al. 2023). Supplementation of the carboxylated form of CBD, cannabidiolic acid (CBDA), and its precursor cannabigerolic acid (CBGA) into cabbage looper diets (*Trichoplusia ni*) also reduced insect growth and increased mortality (Stack et al. 2023).

Treatment with CBD oil on post-harvest wheat (*Triticum turgidum*), corn (*Zea mays*), and rice (*Oryza sativa*) led to a higher mortality rate than neem oil for confused flour beetle (*Tribolium confusum*) and sawtoothed grain beetle (*Oryzaephilus surinamensis*) (Mantzoukas et al. 2020). Extracts of cannabis have also been reported to have insecticidal properties (McPartland 1997; McPartland 2018; Ona et al. 2022). Multiple cannabis extracts were found to have activity against different aphid species. For example, cannabis extracts negatively impacted greenbug (*Schizaphis graminum*) (Chermenskaya et al. 2010) and cabbage aphids (*Brevicoryne brassicae*) mortality (Ahmed et al. 2020). Essential oils derived from hemp were highly toxic and negatively impacted green peach aphid (*Myzus persciae*) mortality (Benelli et al. 2018a). The insecticidal properties of cannabis extracts extend past agricultural pests and have been shown as potential ways to control multiple mosquito species (Maurya et al. 2008; Wanas et al. 2020; Rossi et al. 2020; Bedini et al. 2016; Benelli et al. 2018a, b; Rodriguez et al. 2024). The primary active compound of one of these extracts that was toxic to mosquitoes was identified to be CBD (Rodriguez et al. 2024).

Cannabis aphids (*Phorodon cannabis*) are one of the most damaging pests to hemp and have been identified throughout North America (Cranshaw et al. 2019). Cannabis aphid feeding can cause chlorosis, stunting, and reduced plant vigor. They also generate indirect damage acting as viral vectors for plant viruses (McPartland 1999; McPartland, 2000; Pitt et al. 2022). Cannabis aphids are specialists on *C. sativa*, and the supplementation of CBD into an artificial diet did not impact aphid survival and, in contrast, led to an increase in fecundity (MacWilliams et al. 2023). However, cannabis aphid lifespan was shorter (5.31 ± 0.94 days compared to 10.19 ± 0.9 days), and fecundity was lower (15.54 ± 3.28 nymphs compared to 39.67 ± 4.83 nymphs) in the high-cannabinoid hemp genotype compared to the low-cannabinoid hemp genotype in whole-plant assays. This suggests that other cannabinoids and not CBD may be involved in plant defense against cannabis aphids (MacWilliams et al. 2023). Further analysis of the cannabinoids in the high- and low-cannabinoid hemp genotypes revealed that the minor cannabinoid cannabidivarinic acid (CBDVA), was significantly higher in the high-cannabinoid genotype compared to the low-cannabinoid genotype and may be the contributing factor for decreased aphid performance in whole-plant assays.

**Fig. 1:**
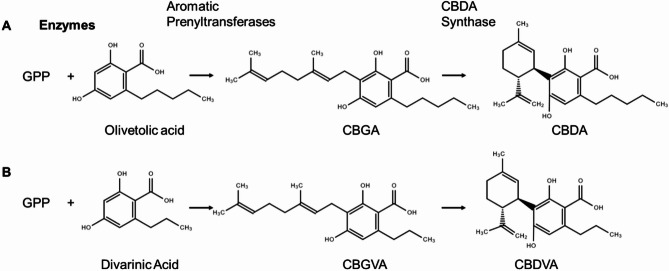
Comparison of the olivetolic and divarinic cannabinoid biosynthesis pathways. The biosynthesis pathways for (**A**) cannabidiolic acid (CBDA) and (**B**) cannabidivarinic acid (CBDVA). Both synthesis pathways begin with geranyl diphosphate (GPP) and (**A**) olivetolic acid or (**B**) divarinic acid through aromatic prenyltransferase activity. CBDA synthase then converts (**A**) cannabigerolicacid (CBGA) to cannabidiolic acid (CBDA) or (**B**) cannabigerovarinic acid (CBGVA) to cannabidivarinic acid (CBDVA), respectively. Chemical structures were drawn using ChemSpider

Cannabinoid biosynthesis has two major precursor pathways: the polyketide pathway and the methylerythritol 4-phosphate (MEP) pathway (Tahir et al. 2021). The polyketide pathway starts from a fatty acid derivative, providing the phenolic functional group of the cannabinoid. Typically, the fatty acid derivative with hexanoic acid is converted to hexanoyl-CoA by an acyl-activating enzyme (AAE) (Stout et al. 2012). Hexanoyl-CoA is then condensed with 3x malonyl-CoA molecules with a polyketide synthase (PKS) followed by cyclization by olivetolic acid cyclase (OAC) to form olivetolic acid (OA) (Taura et al. 2009; Gagne et al., 2012). The MEP pathways lead to the production of geranyl diphosphate (GPP) (Fellermeier et al. 2001). An aromatic prenyltransferase (APT) then adds terpenoid prenyl group from GPP to OLA to form cannabigerolic acid (CBGA) (Fellermeier and Zenk 1998). CBGA is the main precursor of cannabinoid synthesis and produces CBDA or tetrahydrocannabinolic acid (THCA) following through the cyclization of a prenyl moiety with the aid of specific synthases (Fig. 1A) (Taura et al. 1995, 2007, 2009; Fellermeier and Zenk 1998). In contrast to the three major cannabinoids (CBGA, CBDA, and THCA), there are minor cannabinoids that are present at low concentrations in the plant (ElSohly and Gul, 2014; Andre et al. 2016; Hanuš et al. 2016; Berman et al. 2018; Laaboudi et al. 2024; Walsh et al. 2021). One such minor cannabinoid is CBDVA, which is synthesized in the same manner but with the substitution of OA with divarinic acid (DA) (Fig. 1B). This substitution changes the carbon from a pentyl- group to a propyl-group (Vollner et al. 1969; Gill et al. 1970; Taura et al. 2009). CBDVA is a relatively understudied cannabinoid, and only recently has it been identified to have anticonvulsant properties and the potential for use in epilepsy treatments (Anderson et al. 2021). The role of these minor cannabinoids on pest resistance is unknown. In the current study, we sought to elucidate the adaptive role of CBDVA in plant defense against cannabis aphids. The following objectives accomplished this: (1) determine cannabis aphid population growth on high- and low-CBDVA hemp genotypes, and (2) determine the impact of CBDVA supplementation on survival and fecundity of cannabis aphids and the generalist, non-adapted green peach aphid (*M. persicae*).

## Methods

### Aphid and plant sources

Cannabis aphids were collected from an indoor hemp facility in Loveland, Colorado, and maintained on a low-cannabinoid hemp cultivar ‘Elite’ since 2019. ‘Elite’ is an AOSCA-certified low-CBD cultivar (2–3%, R. Fletcher, New West Genetics personal communication) or grain and fiber cultivar and was grown from seed. The aphids are kept on these plants year-round and by maintaining them on a single genotype this minimizes the exposure/adaptation to cannabinoids. The aphid colony is maintained in 45.7 × 45.7 × 76.2 cm cage (BioQuip Products Inc., Rancho Dominguez, CA) under the following greenhouse conditions: 430 W HPS (High Pressure Sodium) Fixtures (P.L. Light Systems) and 400 W bulbs (GE Lucalox lu400 series), 16:8 (L: D) hours (h) photoperiod and the day and night temperatures were 23 °C and 18 °C, respectively. The light intensity was about 200 lum/sq ft with about 30% humidity.

Hemp genotypes used in this study were obtained from the USDA-ARS Hemp Germplasm Collection at the Plant Genetic Resources Unit in Geneva, New York, within the USDA Agricultural Research Service’s National Plant Germplasm System (NPGS). The genotypes were chosen based on their reported CBDVA % dry mass. The high-CBDVA genotypes were G 33,212 ‘S1’ (0.20% CBDVA/dry mass), G 33,296 ‘Carolina Dream’ (0.19% CBDVA/dry mass), and G 33,271 ‘WI-M-H-19-00100’(0.15% CBDVA/dry mass). The low-CBDVA genotypes were G 33349 ‘Carmagnola OP’ (0.04% CBDVA/dry mass) G 33272 ‘WI-M-H-19-00101’ (0.03% CBDVA/dry mass), and G 33273 ‘WI-M-H-19-00102’ (0.02% CBDVA/dry mass). All hemp genotypes were grown from seed. The plants were grown in a greenhouse at Colorado State University’s Plant Growth Facilities under the above-mentioned conditions. Plants were fertilized with Osmocote (Scott’s Company, Marysville, OH) 15-9-12 N: P:K ratio time-released fertilizer as per label instructions and watered ad libitum.

### Assessing aphid populations on high- and low-CBDVA genotypes

To determine the impact of CBDVA on cannabis aphid performance, aphid populations were monitored on high- (S1, Carlonia Dream, and WI-M-H-19-00100) and low-CBDVA (Carmagnola OP, WI-M-H-19-00101, and WI-M-H-19-00102) genotypes. Each genotype was grown in separate insect mesh cages (55 cm x 60 cm x 165 cm) constructed with PVC pipes. Five adult aphids were placed on each of the second uppermost leaf (fully expanded), approximately 10 cm sq, of a 7-week-old hemp plant. The total number of aphids were counted after 7, 10 and 14 days.The experiment was repeated twice with a total number of the following replicates per genotype: S1 (*n* = 12), Carlonia Dream (*n* = 9), WI-M-H-19-00100 (*n* = 8), Carmagnola OP (*n* = 10), WI-M-H-19-00101 (*n* = 7), WI-M-H-19-00102 (*n* = 7). Experiments were performed under the greenhouse conditions listed above.

### Trichome density and morphology in high- and low-CBDVA genotypes

To determine trichome density and morphology in the different genotypes, the middle sections of the middle leaflet of the 2nd to 5th node on 7-week-old plants were observed with a Dino-Lite AM4113T (Dunwell Tech, Inc., Dino-Lite US, Torrance, CA handheld microscope at 225X magnification for S1 and WI-M-H-19-00102 genotypes. Images per plant were captured from the middle portion of the leaves and five to seven images were taken for each leaf of each plant for each genotype. The trichomes on the main meristem between the 1st and 4th nodes were also observed under the same conditions. Each genotype had 6 to 7 images taken for each node. Trichome density and length were calculated using ImageJ software (1.46r).

### Assessing aphid performance with CBDVA supplementation in artificial feeding assays

To determine the role of CBDVA in aphid performance, cannabis aphids and green peach aphids were reared on an artificial diet supplemented with CBDVA dissolved in DMSO. The artificial diet was designed for green peach aphids (Mittler and Dadd 1966). Ten age synchronized 1-day old adult aphids were placed in an artificial feeding chamber consisting of 55 mm diameter Petri dishes (VWR) with parafilm (Bemis) as described in MacWilliams et al. 2023. The volume of the artificial diet was 250 µL including the spiked in CBDVA/DMSO, or DMSO. Stock CBDVA (Cayman Chemical) was prepared by dissolving 1 mg in 30.3 µL of DMSO to make 100 mM CBDVA. The same volume of DMSO was used in the DMSO controls as the highest concentration of cannabinoid screened (2.5 µL for cannabis aphids, 1.25 µL for green peach aphids). Aphids were monitored daily for 3–4 days. There were between 9 and 14 replicates per treatment.

### Analysis of CBDVA using UV detector

Female flower tissue samples for cannabinoid analysis were obtained from the high- (S1) and low- (WI-M-H-19-00102) CBDVA hemp genotypes. The uppermost flower was collected from 14-week-old plants in aluminum envelopes before being frozen in liquid nitrogen. Samples were maintained at -80 °C until they were lyophilized for 72 h. Lyophilized samples were then homogenized for 5 min using a bead beater (Next Advance, Troy, NY, USA). After homogenization, 13 ± 3 mg of tissue for each sample was weighed into 2 ml Eppendorf tubes. An amount of 2 mL of 200-proof ethanol was added to each sample, and the samples were sonicated at 40 kHz in a sonicator bath (Branson Ultrasonics, St. Louis, MO, USA) for 30 min. Following sonication, the samples were protected from light and allowed to sit overnight at room temperature. The samples were then filtered through a 0.45-µm Nylon or PTFE membrane filter into an LC sample vial for analysis.

The cannabinoid analysis was performed on a Thermo Scientific Dionex UltiMate 3000 (Waltham, MA, USA) UPLC system. Mobile phase A consisted of 18 MΩ ultrapure water with 5 mM ammonium formate and 0.10% formic acid, and mobile phase B consisted of LC-MS grade acetonitrile with 0.10% formic acid. The autosampler chamber and column temperatures were 8 °C and 10 °C, respectively, and the cannabinoids were measured at 228 nm. One microliter of sample was injected and separated through a Supelco, Inc. (Burlington, MA, USA) Ascentis^®^ Express 90 Å C18, 2 μm, 15 cm × 2.1 mm column at a flow rate of 0.350 mL/min. We applied the following gradient parameters: 0–2 min, increased to 90% B from 75% B (convex curve 1); 2–5 min, held at 90% B; 5–5.1 min, increased to 100% B (linear curve 5); 5.1–6.1 min, held at 100% B; 6.1–6.2 min, decreased to 75% B (linear curve 5); and 6.2-8, held at 75% B followed by 2.0 min of equilibration at 75% B between injections. The total run time for the instrument was 10 min. Peak areas (mAU/min) were detected and quantified in Chromeleon 7 (v7.2) using the Cobra algorithm. “Auto range” was checked under the baseline noise range and the Cobra smoothing width was set to “auto” [min]. Inhibit integration was “on” from 0 to 1.50 min, then set to “off” from 1.50 to 7.00 min, and turned back “on” at 7.00 min. At 1.50 min, the minimum area was set to “auto.” There were 5 replicates for WI-M-H-19-00102 and 6 replicates for S1.

### Statistical analysis

Statistical analysis was performed using GraphPad Prism version 10.0.0 for Windows, GraphPad Software, Boston, Massachusetts USA. The aphid populations on high and low-CBDVA genotypes were analyzed with Kruskal-Wallis followed by Dunn’s post-hoc test for each timepoint. Trichome density and morphology were analyzed using an unpaired one-tailed t-test. Aphid artificial feeding was analyzed using a one-way ANOVA followed by Tukey’s HSD test for each timepoint. CBDVA amounts were analyzed with a Mann-Whitney test.

## Results

### Cannabis aphid population is negatively impacted on high-CBDVA hemp genotypes

**Fig. 2:**
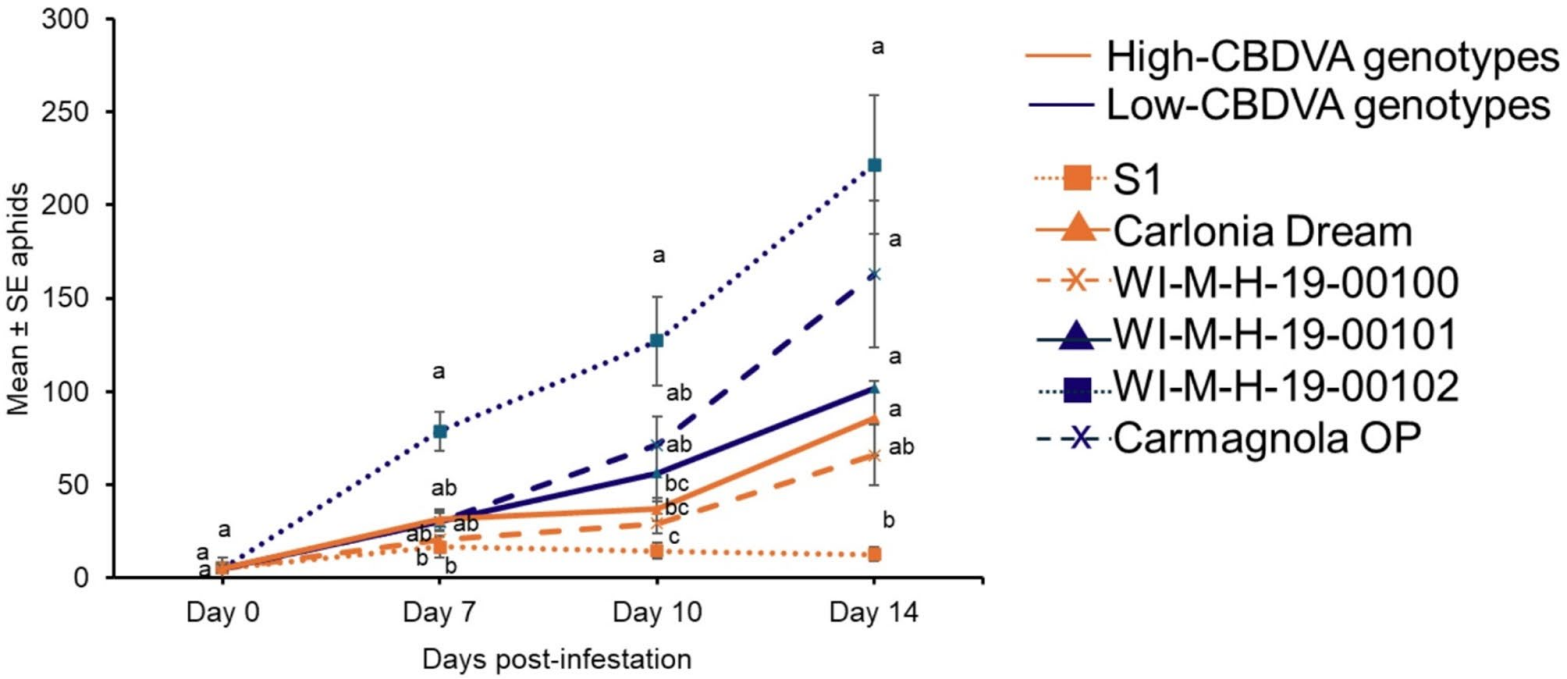
Cannabis aphid population growth on high- and low-cannabidivarinic acid (CBDVA) hemp genotypes. Five adult cannabis aphids were placed on 7-week-old plants and the population was monitored for two weeks. Carmagnola OP, WI-M-H-19-00101, WI-M-H-19-00102 are low-CBDVA genotypes and Carolina Dream, S1, and WI-M-H-19-00100 are high-CBDVA genotypes. The aphid counts at each time-point were analyzed with Kruskal-Wallis followed by Dunn’s post-hoc test. Different letters indicate significance at *P* < 0.05

Cannabis aphid populations were monitored on high-CBDVA genotypes (Carolina Dream, S1, and WI-M-H-19-00100) and low-CBDVA genotypes (Carmagnola OP, WI-M-H-19-00101, WI-M-H-19-00102) under greenhouse conditions. After 7 days, there was a significant difference in the number of aphids observed among the genotypes (Fig. 2; Supplemental Table 1). The population more than doubled in the low-CBDVA (WI-M-H-19-00102) genotype compared to the two high-CBDVA (WI-M-H-19-00100 and S1) genotypes (Fig. 2). After two weeks, the aphid population in the low-CBDVA genotype (WI-M-H-19-00102) was more than 17-fold greater than the high-CBDVA (S1) genotype. The other two low-CBDVA genotypes, WI-M-H-19-00101 and Carmagnola OP had a significantly higher population than S1 after two weeks (Fig. 2; Supplemental Table 1).

The CBDVA levels in the mature flowers were confirmed on the two genotypes in which the aphids performed the best (WI-M-H-19-00102) and the worst (S1). The CBDVA levels in S1 were 1.46 mg/g or 0.13% dry weight, which was significantly higher (*U* = 2, *P* = 0.0087) compared to WI-M-H-19-00102, with 0.21 mg/g and 0.02% dry weight.

### Trichome density and morphology varied between the high- and low-CBDVA genotypes

**Fig. 3:**
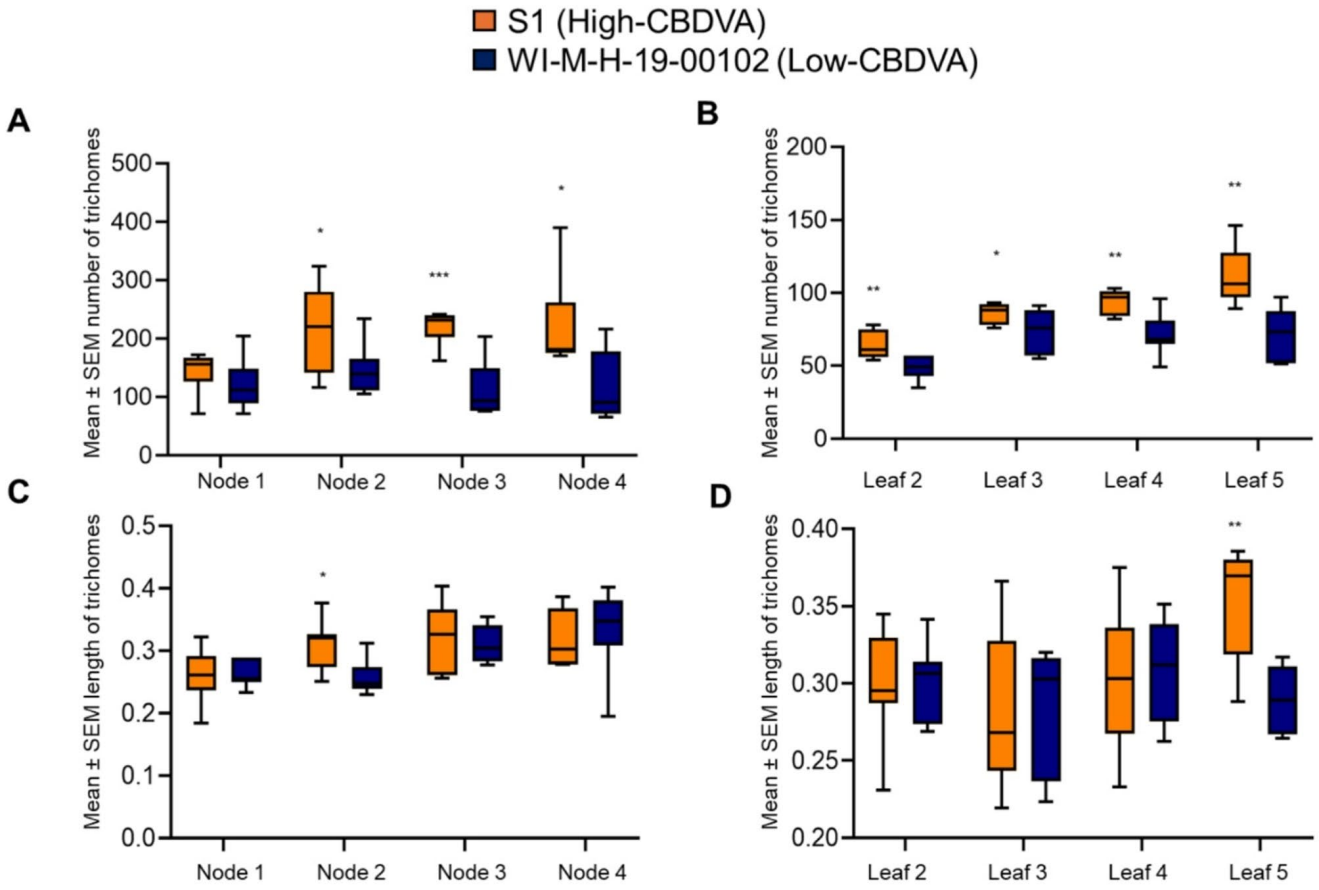
Trichome density and length in high- and low-cannabidivarinic acid (CBDVA) hemp genotypes. The differences observed in trichomes on high- (S1) and low- (WI-M-H-19-00102). (**A**) The number of trichomes observed on meristems, (**B**) the number of trichomes observed on leaves, (**C**) the length of trichomes observed on meristem, and (**D**) the length of trichomes observed on leaves. Images of the meristem between nodes as well as the middle section of the middle leaflet were taken with a Dino-Lite AM4113T at 225X magnification The trichome density and trichome length were done using ImageJ. The density and length were analyzed with unpaired one-tailed t-test. Asterisks indicate significance, * *P* < 0.05, ** *P* < 0.01, *** *P* < 0.005

The two genotypes with the greatest aphid population differences (S1 and WI-M-H-19-00102) had significantly different trichome density and morphology (Supplemental Fig. 1). There were significantly more trichomes in the high-CBDVA genotype (S1) than in the low-CBDVA genotype (WI-M-H-19-00102) at each node after the first node (Fig. 3A; Supplemental Table 2). The trend of increased trichome numbers on S1 compared to WI-M-H-19-00102 was also observed on the leaves (Fig. 3B; Supplemental Table 2). Minimal differences were observed in trichome lengths between the two genotypes with only a significant difference observed on the meristem at node 2 (t = 2.49, *P* = 0.014) (Fig. 3C).

### CBDVA has a detrimental impact on cannabis aphids in artificial feeding assays

**Fig. 4:**
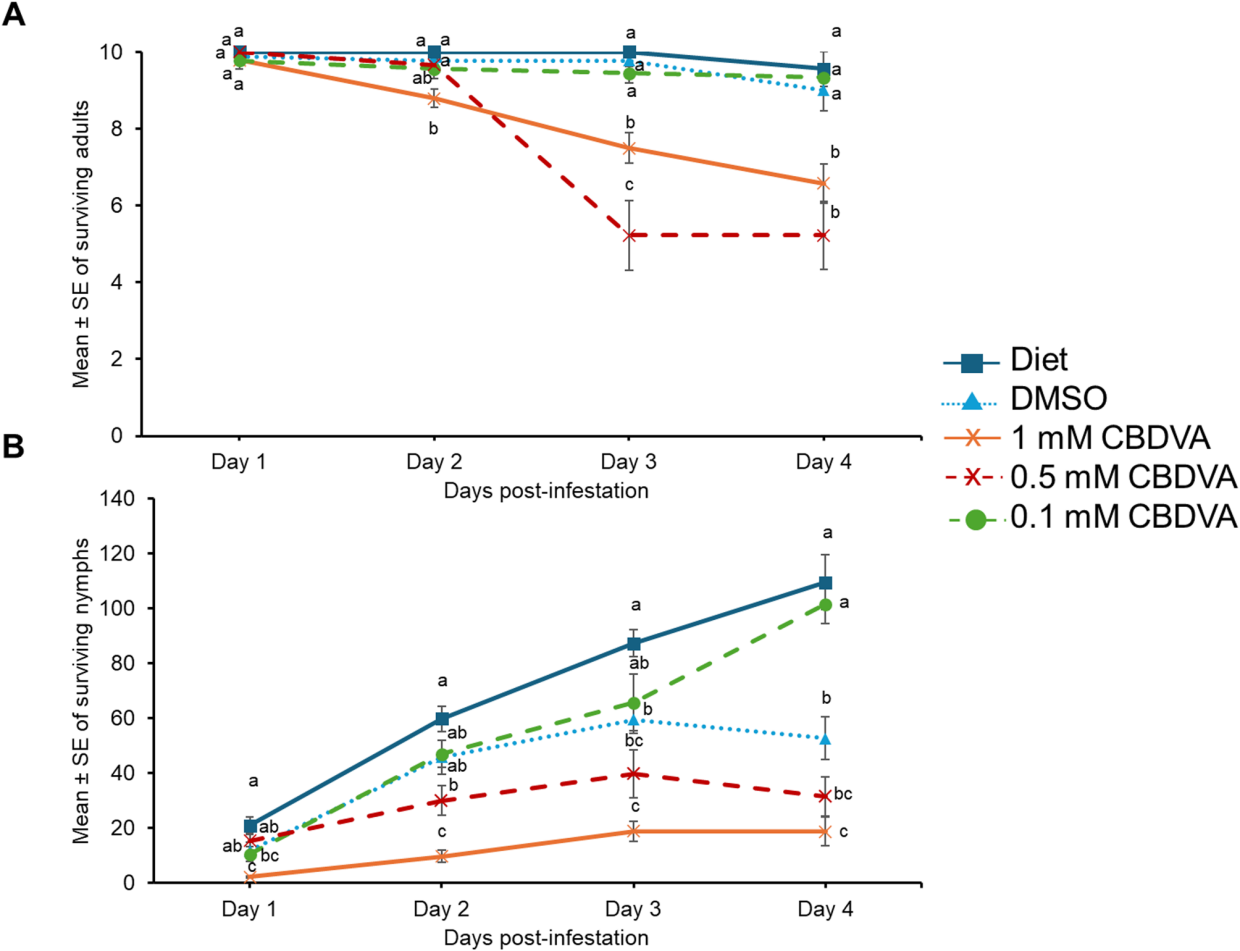
CBDVA has detrimental effects on cannabis aphids in artificial feeding assay. Cannabis aphids were maintained on artificial diet supplemented with either DMSO, 1 mM CBDVA + DMSO, 0.5 mM CBDVA + DMSO, or 0.1 mM CBDVA + DMSO. A cohort of ten 8-day old adult aphids were transferred to artificial diet and allowed to feed for 4 days and the number of and (**A**) surviving adults and (**B**) nymphs remaining each day was monitored (*n* = 9 diet, *n* = 9 DMSO, *n* = 14 for 1 mM CBDVA, *n* = 9 for 0.5 mM CBDVA, and *n* = 9 for 0.1 mM CDBVA). The aphid counts at each time-point were analyzed with one-way ANOVA followed by Tukey’s HSD post-hoc test. Different letters indicate significance at *P* < 0.05

The effect of CBDVA was determined by supplementing CBDVA in artificial aphid diets as previously described (MacWilliams et al. 2023). The diet supplemented with 1 mM CBDVA + DMSO had significantly less aphid survival as early as day 2 compared to DMSO and diet controls (Fig. 4A; Supplemental Table 3). On days 3 and 4, 0.5 mM CBDVA + DMSO also had detrimental effects on adult survival compared to DMSO and diet only controls. The 0.1 mM CBDVA + DMSO supplemented diets did not impact adult aphid survival at any time point (Fig. 4A).

Cannabis aphid fecundity was significantly reduced starting as early as day 1 in the diet supplemented with 1 mM CBDVA + DMSO compared to DMSO and diet controls (Fig. 4B; Supplemental Table 3). This trend was observed for the remainder of the experiment. The diet supplemented with 0.5 mM CBDVA + DMSO also reduced fecundity compared to diet control starting at day 2, but there was no difference between 0.5 mM CBDVA + DMSO supplemented diet and the DMSO control. Only on day 1, a significant difference was observed between diet supplemented with 0.1 mM CBDVA + DMSO compared to diet control.

### CBDVA has a detrimental impact on green peach aphids in artificial feeding assays

**Fig. 5:**
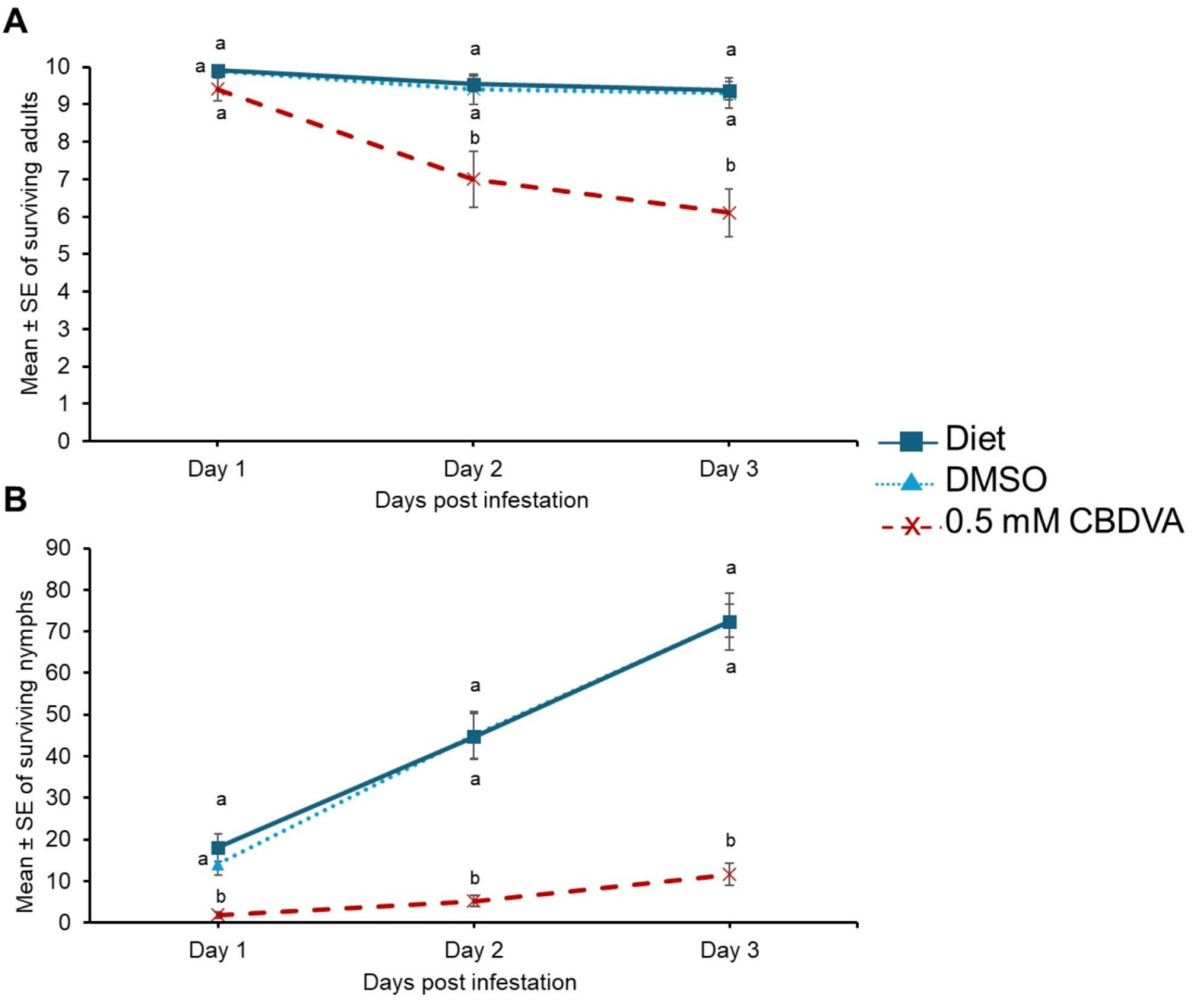
CBDVA has detrimental effects on green peach aphids in artificial feeding assay. Green peach aphids were maintained on an artificial diet supplemented with either DMSO, or 0.5 mM CBDVA + DMSO. A cohort of ten 8-day old adult aphids were transferred to artificial diet and allowed to feed for 3 days and the number of (**A**) surviving adults and (**B**) remaining each day was monitored (*n* = 10). The aphid counts at each time-point were analyzed with one-way ANOVA followed by Tukey’s HSD post-hoc test. Different letters indicate significance at *P* < 0.05

Green peach aphid survival on diet with supplemented 0.5 mM CBDVA + DMSO was significantly reduced on day 2 and 3 compared to DMSO and diet controls (Fig. 5A; Supplemental Table 4). The negative impact of CBDVA was observed as early as day 1 with a significant reduction as in the diet supplemented with 0.5 mM CBDVA + DMSO compared to DMSO and diet controls (Fig. 5B; Supplemental Table 4). This trend was consistent for days 2 and 3.

## Discussion

*Cannabis sativa* has a wealth of specialized metabolites, including cannabinoids and terpenes, which are known to be involved in the plant’s defense mechanism against arthropod pests (McPartland 1997). To date, most studies have focused on the role of major cannabinoids such as CBDA, CBD, THCA, and THC in defense against insects (Rothschild, 1977; Rothschild and Fairborn, 1980; Park et al. 2019; Abendroth et al. 2023). Here, we demonstrated the potential insecticidal properties of a naturally occurring minor cannabinoid, CBDVA, in plant defense against the cannabis-adapted specialist aphid, cannabis aphids (*P. cannabis*), and the non-adapted generalist, green peach aphid (*M. persicae*). In a previously published paper, we reported that cannabis aphid lifespan was shorter, and fecundity was lower in a high-cannabinoid hemp genotype compared to the low-cannabinoid hemp genotype in whole-plant assays. Analysis of cannabinoid levels from the leaves in the high- and low-cannabinoid genotypes revealed that CBDVA was significantly higher in the high-cannabinoid genotype compared to the low-cannabinoid genotype, and there was no difference in levels of other cannabinoids, including THC and CBD. This led us to hypothesize that other cannabinoids could be contributing to the reduced aphid performance on the high-cannabinoid cultivar and not CBD or THC (MacWilliams et al. 2023).

The current study evaluated a panel of hemp genotypes from the USDA Agricultural Research Service’s National Plant Germplasm System (NPGS) differing in CBDVA levels. We found that cannabis aphids performed poorly on the high-CBDVA genotypes compared to low-CBDVA genotypes. Specifically, aphids had the highest population on the low-CDBVA genotype (WI-M-H-19-00102) and lowest population on the high-CBDVA genotype (S1). The cannabinoid levels measured in the female flowers from the current study confirmed the cannabinoid levels reported from the USDA-ARS Hemp Germplasm Collection. However, aphids in the current study were placed on the leaves, not the flowers. To confirm the effect of CBDVA, we performed artificial feeding assays where aphid diets were supplemented with different concentrations of CBDVA. In a previous study, we measured CBDVA levels in leaves, and it was 500 µg/g (1.5 mM) (MacWilliams et al. 2023), which is the range of concentrations 0.1 mM- 1 mM) used in the current study. We confirmed that CBDVA negatively impacted cannabis aphids at all concentrations and the 1 mM CBDVA concentration had the most detrimental effect.

The negative impact of CBDVA was also observed with the generalist green peach aphids in the artificial feeding assays. The presence of CBDVA had a more drastic effect on green peach aphids than cannabis aphids at lower concentrations. We hypothesize that the drastic effects on green peach aphids are likely because they are not adapted to hemp. Cannabinoid production evolutionary is believed to be a plant defense mechanism against plant pests (Stack et al. 2023). The non-adapted green peach aphids are highly susceptible to the presence of cannabinoids. This is most likely due to cannabis aphids evolving and adapting with its host plant in an evolutionary arms race (War et al. 2012).

One of the major physical differences observed between the high- and low-CBDVA genotypes was in trichome density and morphology. Trichomes impact plant defense, acting as a physical barrier to plant pests (War et al. 2012). For example, in chrysanthemum (*Chrysanthemum grandiflorum*), the most resistant cultivar to the chrysanthemum aphid (*Macrosiphoniella sanbourni*) had the longest and densest trichomes (He et al. 2011). The effects of trichomes on aphids go beyond just physical barriers. Potato aphids (*Macrosiphum euphorbiae*) reared on isogenic tomato (*Solanum* spp.) lines that differed by the presence of type IV glandular trichomes increased oxidate stress responses in the aphids on the tomatoes with the type IV glandular trichomes (Planello et al., 2022). The high-CBDVA genotype, S1 had significantly more trichomes than the low-CBDVA genotype, WI-M-H-19-00102, which likely inhibited aphid movement and population growth on the high-CBDVA lines. The molecular mechanisms underlying the effect of CBDVA on aphids are unknown and currently under investigation in our laboratory. Interestingly, insects are one of the few animal groups that do not possess canonical cannabinoid receptors (McPartland et al. 2001, 2006).

The potential use of CBDVA as a pesticide is an exciting area for future research. Unlike CBD and THC, CBDVA is thought to have minimal effects on humans. The anticonvulsant activity of CBDVA was only identified in the highest concentration (100 mg/kg) tested in mice (Anderson et al. 2021). In hippocampal neurons and dorsal root ganglion neurons CBDVA had no effect on signaling (Straiker et al. 2021). The neutral form of CBDVA (CBDV) had low to no binding affinities to the human cannabinoid receptor CB_1_R in in vitro assays. A higher affinity was identified for human cannabinoid receptor CB_2_R but still lower than other cannabinoids (Navarro et al. 2020; Zagzoog et al. 2020). In vivo assays, CBDV produced no significant responses at doses that ranged from 0.1 to 10 mg/kg doses (Zagzoog et al. 2020). CBDV could interfere with cannabinoid signaling postsynaptically (Straiker et al. 2021).

Currently, the majority of the biopesticides identified and used from cannabis are essential oils. Hemp essential oils have been effective against agricultural pests including multiple species of aphids and as insecticide to control mosquitoes (Chermenskaya et al. 2010; Wanas et al. 2020; Rossi et al., 2020; Bedini et al. 2016; Benelli et al. 2018a, b). While they have been shown to be effective, essential oils can vary in their composition (Ona et al. 2022). Hence screening for hemp genotypes with high CBDVA levels and increasing levels through traditional breeding or genetic modification is a way to develop pest-resistant varieties or increase CBDVA production to produce essential oils. While these essential oils are less toxic than conventional pesticides, future research should evaluate effects on non-target arthropods and beneficial insects. An essential oil derived from tea tree (*Melaleuca alternifolia*), to be used against the Asian tiger mosquito (*Aedes albopictus*) was highly effective, however it was also found to be highly toxic to the non-target water flea (*Daphnia magna*) (Conti et al., 2014). Future tests of CBDVA with other pests and beneficial insects are needed to maximize its potential use while minimizing possible off-target effects.

## Conclusions

This study provided evidence that CBDVA is effective against both hemp-specific and generalist aphid pests. These findings indicate that CBDVA may be a strong candidate for naturally derived pest control, particularly in organic farming and in crops such as hemp where chemical pesticides are restricted or limited. Hemp is an emerging, readily cultivated crop in the U.S., and hemp leaves are often discarded. Extracting essential oils from hemp leaves could help provide an additional revenue stream for hemp growers from a previously discarded part of the plant and may provide a sufficient amount of CBDVA and other bioactive compounds to efficiently produce biopesticides at scale. This could expand the market for hemp products and reduce the need for synthetic chemicals in pest control. Future studies should evaluate the extraction feasibility, stability in formulations, and cost-effectiveness of CBDVA as a biopesticide compared to synthetic pesticides.

## Electronic supplementary material

Below is the link to the electronic supplementary material.

Supplementary Material 1

## Data Availability

No datasets were generated or analysed during the current study.

## Abbreviations

THC: Tetrahydrocannabinol
CBD: Cannabidiol
CBDVA: Cannabidivarinic acid
CBDA: Cannabidiolic acid
MEP: Methylerythritol 4-phosphate
AAE: Acyl-activating enzyme
PKS: Polyketide synthase
OAC: Olivetolic acid cyclase
OLA: Olivetolic acid
GPP: Geranyl diphosphate
APT: Aromatic prenyltransferase
CBGA: Cannabigerolic acid
THCA: Tetrahydrocannabinolic acid
