# Supplementary material for: Assessing the adaptive role of cannabidivarinic acid (CBDVA) in aphid defense in Cannabis sativa
Source: J Cannabis Res. 2025 Jun 11;7:34. doi: 10.1186/s42238-025-00291-x (PMC12153158; doi:10.1186/s42238-025-00291-x)
Supplement: Supplementary file 1 — Supplementary Material 1 [file 42238_2025_291_MOESM1_ESM.docx]

**Supplemental Table 1.** Statistical analysis of cannabis aphid populations on high- and low-CBDVA hemp genotypes

|  | **Day 0** | **Day 7** | **Day 10** | **Day 14** |
| --- | --- | --- | --- | --- |
| **S1** | 5.00 ± 0.00^a^ | 16.50 ± 5.56^b^ | 14.17 ± 4.31^c^ | 12.58 ± 3.53^b^ |
| **Carolina Dream** | 5.00 ± 0.00^a^ | 31.11 ± 5.27^ab^ | 36.89 ± 6.21^bc^ | 85.78 ± 20.11^a^ |
| **WI-M-H-19-00100** | 5.00 ± 0.00^a^ | 20.5 ± 2.06^b^ | 29.13 ± 5.13^bc^ | 65.75 ± 16.41^ab^ |
| **WI-M-H-19-00101** | 5.00 ± 0.00^a^ | 30.14 ± 5.80^ab^ | 56.14 ± 4.65^ab^ | 101.71 ± 15.00^a^ |
| **WI-M-H-19-00102** | 5.00 ± 0.00^a^ | 78.57 ± 10.59^a^ | 127.00 ± 23.82^a^ | 221.57 ± 37.27^a^ |
| **Carmagnola OP** | 5.00 ± 0.00^a^ | 30.00 ± 5.11^ab^ | 71.20 ± 15.30^ab^ | 162.90 ± 39.35^a^ |
| **H-statistic** | n/a | 22.67 | 32.76 | 31.04 |
| ***P-value*** | n/a | *0.004* | *<0.0001* | *<0.0001* |

Overall model analysis was performed with Kruskal-Wallis at each time point. Comparisons in each timepoint were performed with Dunn’s post-hoc test. The different letters indicate significance at *P* < 0.05.

**Supplemental Table 2.** Statistical analysis of trichome density on high- and low-CBDVA genotypes

|  | **S1** | **WI-M-H-19-00102** | **t** | ***P*** |
| --- | --- | --- | --- | --- |
| **Leaf 2** | 63.71 ± 3.48 | 48.29 ± 3.08 | 3.32 | ***0.003*** |
| **Leaf 3** | 85.86 ± 2.47 | 72.29 ± 5.57 | 2.23 | ***0.023*** |
| **Leaf 4** | 93.71 ± 3.12 | 71.57 ± 5.61 | 3.45 | ***0.0024*** |
| **Leaf 5** | 111.00 ± 9.42 | 71.67 ± 7.35 | 3.35 | ***0.0043*** |
| **Node 1** | 142.90 ± 13.20 | 122.10 ± 16.73 | 0.97 | *0.18* |
| **Node 2** | 216.40 ± 27.47 | 145.90 ± 16.68 | 2.20 | ***0.024*** |
| **Node 3** | 218.40 ± 10.84 | 111.90 ± 17.84 | 5.11 | ***0.0001*** |
| **Node 4** | 219.80 ± 34.75 | 120.60 ± 22.71 | 2.46 | ***0.016*** |

Analysis was performed using an unpaired one-tailed t-test Bold indicates significant differences between treatments at *P* < 0.05.

|  | **Day 1** | | **Day 2** | | **Day 3** | | **Day 4** | |
| --- | --- | --- | --- | --- | --- | --- | --- | --- |
|  | Adults | Nymphs | Adults | Nymphs | Adults | Nymphs | Adults | Nymphs |
| **Diet** | 10.00 ± 0.00^a^ | 21.00 ± 3.00^a^ | 10.00 ± 0.00^a^ | 59.78 ± 4.49^a^ | 10.00 ± 0.00^a^ | 87.33 ± 5.01^a^ | 9.56 ± 0.44^a^ | 109.60 ± 10.01^a^ |
| **DMSO** | 9.89 ± 0.11^a^ | 11.78 ± 3.04^ab^ | 9.78 ± 0.15^a^ | 45.89 ± 6.15^ab^ | 9.78 ± 0.15^a^ | 59.56 ± 5.10^b^ | 9.00 ± 0.53^a^ | 52.67 ± 7.79^b^ |
| **1 mM CBDVA** | 9.79 ± 0.11^a^ | 2.14 ± 0.43^c^ | 8.79 ± 0.24^b^ | 9.71 ± 2.33^c^ | 7.5 ± 0.40^b^ | 18.79 ± 3.72^c^ | 6.57 ± 0.52^b^ | 18.71 ± 5.21^c^ |
| **0.5 mM CBDVA** | 10.00 ± 0.0^a^ | 15.44 ± 2.37^ab^ | 9.67 ± 0.17^a^ | 30.00 ± 5.41^b^ | 5.22 ± 0.91^c^ | 39.78 ± 8.64^bc^ | 5.22 ± 0.89^b^ | 31.44 ± 7.04^bc^ |
| **0.1 mM CBDVA** | 9.78 ± 0.22^a^ | 10.44 ± 2.71^bc^ | 9.56 ± 0.24^ab^ | 47.00 ± 5.03^ab^ | 9.44 ± 0.24^a^ | 65.78 ± 10.29^ab^ | 9.33 ± 0.24^a^ | 101.70 ± 7.25^a^ |
| **F-statistic** | 0.79 | 10.79 | 6.42 | 21.13 | 17.99 | 17.69 | 11.06 | 32.65 |
| ***P-value*** | *0.54* | *<0.0001* | *0.0004* | *<0.0001* | *<0.0001* | *<0.0001* | *<0.0001* | *<0.0001* |

**Supplemental Table 3.** Statistical analysis of cannabis aphid performance on artificial diets

Overall model analysis was performed with one-way ANOVA at each timepoint. Comparisons in each timepoint were performed with Tukey’s HSD post-hoc test, different letters indicate significance at *P* < 0.05.

|  | **Day 1** | | **Day 2** | | **Day 3** | |
| --- | --- | --- | --- | --- | --- | --- |
|  | Adults | Nymphs | Adults | Nymphs | Adults | Nymphs |
| **Diet** | 10.00 ± 0.00^a^ | 19.40 ± 3.38^a^ | 9.60 ± 0.22^a^ | 47.50 ± 5.40^a^ | 9.40 ± 0.27^a^ | 75.00 ± 6.92^a^ |
| **DMSO** | 9.90 ± 0.10^a^ | 14.10 ± 2.82^a^ | 9.40 ± 0.40^a^ | 45.10 ± 5.60^a^ | 9.30 ± 0.40^a^ | 72.50 ± 7.03^a^ |
| **0.5 mM CBDVA** | 9.40 ± 0.31^a^ | 1.80 ± 0.77^b^ | 7.00 ± 0.75^b^ | 5.20 ± 1.26^b^ | 6.10 ± 0.64^b^ | 11.60 ± 2.60^b^ |
| **F-statistic** | 3.00 | 12.24 | 8.22 | 27.26 | 16.57 | 37.17 |
| ***P-value*** | *0.066* | *0.0002* | *0.0016* | *<0.0001* | *<0.0001* | *<0.0001* |

Overall model analysis was performed with one-way ANOVA at each timepoint. Comparisons in each timepoint were performed with Tukey’s HSD post-hoc test, different letters indicate significance at *P* < 0.05.

**Supplemental Table 4.** Statistical analysis of green peach aphid performance on artificial diets


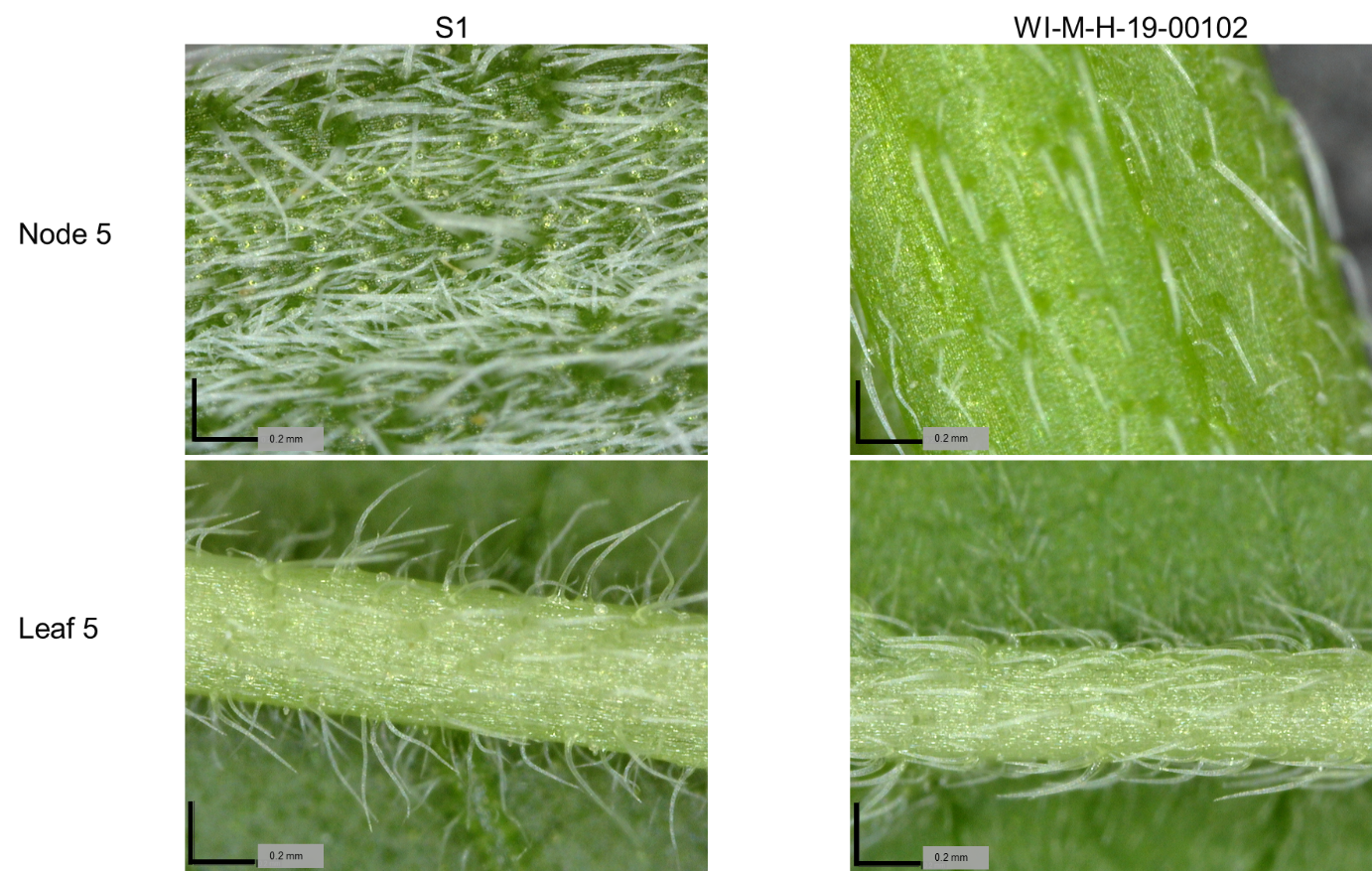


**Supplemental Figure 1.** Observed differences in trichome density and morphology between S1 and WI-M-H-00102 at leaf 5 and node 5 with a Dino-Lite handheld microscope at 225X magnification.
